# Inhibition of hypoxia‐induced Mucin 1 alters the proteomic composition of human osteoblast‐produced extracellular matrix, leading to reduced osteogenic and angiogenic potential

**DOI:** 10.1002/jcp.30617

**Published:** 2021-10-22

**Authors:** Pavitra K. Jadaun, Shuang Zhang, Marijke Koedam, Jeroen Demmers, Suvro Chatterjee, Johannes P. van Leeuwen, Bram C. van der Eerden

**Affiliations:** ^1^ Laboratory for Calcium and Bone Metabolism, Department of Internal Medicine Erasmus University Medical Center Rotterdam The Netherlands; ^2^ Vascular Biology Laboratory, AU‐KBC Research Centre Anna University Chennai Tamil Nadu India; ^3^ Proteomics Centre Erasmus University Medical Center Rotterdam The Netherlands

**Keywords:** angiogenesis, extracellular matrix, human osteoblasts, hypoxia, mucin‐1

## Abstract

The bone microenvironment is one of the most hypoxic regions of the human body and in experimental models; hypoxia inhibits osteogenic differentiation of mesenchymal stromal cells (MSCs). Our previous work revealed that Mucin 1 (MUC1) was dynamically expressed during osteogenic differentiation of human MSCs and upregulated by hypoxia. Upon stimulation, its C‐terminus (MUC1‐CT) is proteolytically cleaved, translocases to the nucleus, and binds to promoters of target genes. Therefore, we assessed the MUC1‐mediated effect of hypoxia on the proteomic composition of human osteoblast‐derived extracellular matrices (ECMs) and characterized their osteogenic and angiogenic potentials in the produced ECMs. We generated ECMs from osteogenically differentiated human MSC cultured in vitro under 20% or 2% oxygen with or without GO‐201, a MUC1‐CT inhibitor. Hypoxia upregulated *MUC1*, vascular endothelial growth factor, and connective tissue growth factor independent of MUC1 inhibition, whereas GO‐201 stabilized hypoxia‐inducible factor 1‐alpha. Hypoxia and/or MUC1‐CT inhibition reduced osteogenic differentiation of human MSC by AMP‐activated protein kinase/mTORC1/S6K pathway and dampened their matrix mineralization. Hypoxia modulated ECMs by transforming growth factor‐beta/Smad and phosphorylation of NFκB and upregulated COL1A1, COL5A1, and COL5A3. The ECMs of hypoxic osteoblasts reduced MSC proliferation and accelerated their osteogenic differentiation, whereas MUC1‐CT‐inhibited ECMs counteracted these effects.

In addition, ECMs generated under MUC1‐CT inhibition reduced the angiogenic potential independent of oxygen concentration. We claim here that MUC1 is critical for hypoxia‐mediated changes during osteoblastogenesis, which not only alters the proteomic landscape of the ECM but thereby also modulates its osteogenic and angiogenic potentials.

## Graphical abstract

Our study claims that Mucin 1 (MUC1) is critical for hypoxia‐mediated changes during osteoblastogenesis that not only alters the proteomic landscape of the extracellular matrice (ECM) but thereby also modulate its osteogenic and angiogenic potentials. The study emphasizes that it is pivotal to understand the role of hypoxia and MUC1 in developing customized ECM scaffolds to induce osteogenesis.

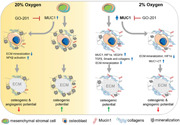

## 1 INTRODUCTION

The extracellular matrix (ECM), a complex of self‐assembled macromolecules, is composed predominantly of collagen, glycoproteins, proteoglycans, and hyaluronan (Trapani et al., 2017). It not only serves as a 3D scaffold for the cells and facilitates their attachment, migration, proliferation, and differentiation but also acts as a reservoir for growth factors and cytokines. In bone, ECM comprises 40% organic (of which 90% constitutes collagen type I, and 10% represents noncollagenous proteins) and 60% inorganic compounds. Moreover, its exact proteomic composition and mineralization vary based on bone type, age, sex, and health conditions (Lin et al., 2020). Similarly, the proteomic composition and mineralization of osteoblast‐secreted ECM can be modulated by altered culture conditions in vitro.

ECM formation with eventual mineralization serves as a hallmark of osteogenic differentiation of mesenchymal stromal cells (MSCs), following deposition and accumulation of proteins to form the ECM, mineralization proceeds, which marks the final phase of the osteoblast phenotypic development (Trapani et al., 2017). The bone ECM coordinates the interaction between osteogenic and angiogenic processes and drives bone growth and tissue restoration during bone repair. Angiogenesis is vital for tissue growth, maturation and repair by facilitating transport of cytokines and growth factors necessary for cell viability, proliferation, and interaction.

Hypoxia is a relevant situation for bone as the tissue is permanently hypoxic (Rankin et al., 2011) and as a consequence, the behavior of bone cells is subject to changes in ambient oxygen concentrations. MSCs are self‐renewable and can differentiate into osteoblasts, fibroblasts, myocytes, and adipocytes (Ankrum et al., 2014). Previous studies including our own have shown that hypoxia inhibits osteogenic differentiation of MSCs and thus osteoblast‐mediated ECM mineralization (Hsu et al., 2013; Nicolaije et al., 2013). Therefore, it is of primary interest to consider oxygen concentration as an important factor in tissue growth and repair for ECM mineralization and neovascularization. Hypoxia‐inducible factors (HIFs) are the primary signaling molecules sensing oxygen concentration, which upon activation trigger several signaling cascades involving vascular endothelial growth factor (VEGF), transforming growth factor‐beta (TGFβ), AMP‐activated protein kinase (AMPK), and nuclear factor‐κB (NF‐κB) (Guo et al., 2016; Wang et al., 2007; D'Ignazio & Rocha, 2016).

Mucin 1 (MUC1) is a transmembrane heterodimeric glycoprotein expressed on the surface of secretory epithelial, mucosal, and hematopoietic cells (Nath & Mukherjee, 2014). We previously found that *MUC1* is upregulated in differentiating osteoblasts derived from human MSC and that Muc1 plays an age‐dependent role in murine bone development (Brum et al., 2018). The extracellular domain of MUC1 (N‐terminal) is composed of a variable number of 20 amino acid tandem repeats with extensive O‐glycans (Dhar & McAuley, 2019). The cytoplasmic tail of MUC1 (MUC1‐CT) consists of a 58 amino acid extracellular domain, a 28 amino acid transmembrane domain, and a 72 amino acid cytoplasmic domain (Nath & Mukherjee, 2014). Upon stimulation, a part of the C‐terminus is proteolytically cleaved, forms a homodimer, and translocases to the nucleus by a mechanism involving importin‐β and Nup62 (Leng et al., 2007). *MUC1* is overexpressed in a number of adenocarcinomas during malignant transformation and it is associated with a protective barrier function at the epithelial cell‐cell surface (Kufe, 2009). Furthermore, it was suggested that MUC1 expression is directly correlated with hypoxia‐induced tumor progression and metastatic urothelial carcinoma in bone (Kaira et al., 2012; Zanetti et al., 2011). Emerging evidence suggests that HIF‐1α directly regulates MUC1 expression under hypoxia in lung and renal carcinoma (Aubert et al., 2009; Mikami et al., 2009;). Previously, we discovered *MUC1* among one of the top differentially modulated genes by hypoxia in a transcriptomic dataset of human osteoblasts (Nicolaije et al., 2013).

Despite the previously generated knowledge regarding the role of MUC1 in bone in vivo (Brum et al., 2018), there is a large knowledge gap as to how and what physiological changes in the ECM are modulated by MUC1 in human osteoblasts? Considering the upregulation of MUC1 under hypoxia and the oxygen concentration is a major modulating factor for ECM and its mineralization, we assessed the role of MUC1 and hypoxia on the proteomic composition of human osteoblast‐produced ECM and its consequences for osteogenesis and angiogenesis.

## 2 MATERIALS AND METHODS

### 2.1 Cell culture

Human bone marrow‐derived MSC were obtained from Lonza (PT‐2501) from a healthy male donor. MSC were cultured at a density of 5000 cells/cm^2^ in a growth medium formulated with Dulbecco's modified Eagle medium (DMEM) (GIBCO) supplemented with 10% heat‐inactivated fetal bovine serum (FBS) (GIBCO), 100 U/ml penicillin and 100 μg/ml streptomycin (Sigma), pH 7.5, for 2 days at 5% carbon dioxide (CO_2_) and 37°C. Subsequently, MSC were osteogenically differentiated by adding 10 nM dexamethasone and 10 mM β‐glycerophosphate (Sigma) to the growth medium. Hypoxic conditions for MSC cultures were created using a multi‐gas incubator (Bionex Solutions Inc.) containing a gas mixture composed of 93% nitrogen, 5% CO_2,_ and 2% oxygen. In some of the experiments, MSC were treated with 5 µM GO‐201 (G7923; Sigma), a specific MUC1 inhibitor, during the course of osteoblast differentiation in both normoxic and hypoxic conditions. Cells were refreshed twice a week. Human umbilical vein endothelial cells (HUVECs) were purchased from American Type Culture Collection and cultured in Dulbecco's modified Eagle medium (DMEM) supplemented with 10% FBS, 100 U/ml penicillin, and 100 μg/ml streptomycin at 5% CO_2_ and 37°C. HUVECs were used between the third and tenth passages for the experiments.

### 2.2 Production of lentiviral particles and lentiviral transduction

HEK293T cells cultured in DMEM medium containing 10% heat‐inactivated FBS and 100 Units/ml penicillin and 100 µg/ml streptomycin were transfected with 600 µl DNA transfection solution (ViraPower, 5 mM HEPES and 125 µM CaCl_2_). The pLKO.1 plasmid inserted with either a specific short hairpin RNA (shRNA) sequence targeted against MUC1 or a random sequence as a control (scrambled) was added to the transfection solution (Table S1). The Virapower system consists of three different plasmids encoding essential viral proteins to form a replication‐deficient lentiviral particle. Twenty‐four hours later, the medium was refreshed with 8 ml 10% DMEM medium containing 20 mM HEPES, and the next day, the virus‐containing medium was collected and filtered through a 0.45 µm filter and directly used for transduction. MSC cultured in 12‐well plates were transduced with MUC1 shRNA‐expressing lentivirus in a serum‐free growth medium and incubated overnight. MSC cultured for 10 days posttransduction were harvested for messenger RNA (mRNA) isolation and MUC1 expression was assessed using RT‐PCR. In another experiment, ECM mineralization of MSC cultured under 20% (normoxic) or 2% O_2_ (hypoxic) for 17 days was assessed by measuring calcium content.

### 2.3 Protein extraction and western blotting

Confluent monolayers of osteogenically differentiating MSC for 11 days were scraped in ice‐cold phosphate buffered saline (PBS), pelleted, and lysed in RIPA buffer (R0278; Sigma) with proteases and phosphatase inhibitors. Proteins in the supernatant were quantified with the Pierce^TM^ BCA assay (Thermo Fisher Scientific). For western blotting, equal amounts of proteins of each sample were boiled with 6X sample buffer (240 mM Tris pH 6.8, 40% glycerol, 8% SDS, 0.002% bromophenol blue, 0.002% β‐mercaptoethanol) for 5 min. The proteins were separated by 12% sodium dodecyl sulfate‐polyacrylamide gel electrophoresis, transferred to nitrocellulose membranes, blocked for 1 h with 5% nonfat milk, and incubated overnight at 4°C with primary antibodies (Table S2). Then, the membranes were incubated with peroxidase‐conjugated donkey anti‐rabbit immunoglobulin G (IgG) (dilution 1:25,000; Thermo Fisher Scientific) or mouse IgG kappa binding protein (dilution 1:1000, Santa Cruz Biotechnology). The signals were enhanced with chemiluminescence reagents (Amersham ECL Prime; GE Healthcare), and quantified with a Fusion camera and its Capt Fx Software (Vilber‐Lourmat).

### 2.4 Devitalization

Devitalization is a process of removal of cytoplasm and nuclear material from cell cultures to obtain an ECM that was previously laid down by the cells. MSC cultured for 11 days under normoxic or hypoxic conditions ± GO‐201 were devitalized as described before (Baroncelli et al., 2018). In brief, the culture medium was removed from osteogenically differentiating MSC and cells were washed twice with phosphate buffered saline (PBS) (Gibco BRL). Cells were subjected to 3X freeze‐thaw cycles followed by DNase treatment for 30 min at 37°C (10 U/ml). Finally, DNase solution was removed and cells were washed extensively using PBS. The matrices were air‐dried and stored at −20°C until further use.

### 2.5 Alkaline phosphatase (ALP) activity and ECM mineralization assays

ALP activity was measured at 6, 11, and 19 days of culture, and matrix mineralization at 6, 11, and 19 days of culture, as previously described (Bruedigam et al., 2011). A protein assay was performed on the same samples to correct for cell numbers as described before (Bruedigam et al., 2011).

### 2.6 Proteomic profiling of ECM using mass spectrometry (MS)

The proteomic composition of the ECM was determined using a label‐free quantification (LFQ) method using MS. ECM samples were collected in PBS and processed for in‐solution trypsin digestion. Then, samples were processed for MS and analyzed as previously described (Baroncelli et al., 2018). Raw MS data were analyzed by using MaxQuant Software (version 1.5.6.0) and the Andromeda search engine and annotated against the human proteome as provided by the Uniprot database (taxonomy: Homo sapiens, release HUMAN_2016_10.0). Samples were run in duplicate and then averaged for analysis.

### 2.7 Bioinformatics analysis

Scatter plots were prepared using the log2 LFQ values of peptide intensity by Perseus v1.3.0.4 (Tyanova et al., 2016). Functional pathway analysis was performed by QIAGEN's Ingenuity® Pathway Analyser (IPA®, QIAGEN Redwood City www.qiagen.com/ingenuity). Proteins uniquely present in a group or fold change ≥2 in the comparison between two groups were used as input. Fisher's exact test with Bonferroni correction (*p* value ≤0.05) was used to identify statistically significant pathways (Bland & Altman, 1995; Fisher, 1992).

### 2.8 Quantitative measurement of gene expression

Total RNA was extracted at Days 1, 6, 11, and 19 of MSC cultures or at Day 5 of HUVEC cultures using TRIzol® reagent (Invitrogen) according to manufacturer's instructions. RNA was isolated as described before (Bruedigam et al., 2011) and quantified by a NanoDrop ND‐1000 spectrophotometer (NanoDrop Technologies, Inc.). The obtained messenger RNA (mRNA) was reverse‐transcribed to complementary DNA (cDNA) using a cDNA synthesis kit according to the manufacturer's instructions (Applied Biosystems). Gene expression was evaluated by RT‐PCR on a QuantStudio 7 Flex Real‐Time PCR System (Applied Biosystems), using SYBR Green PCR master mix reagents (VWR International). All primers sets overspanning at least one intron‐exon boundary are provided in supplementary table 3 (Table S3). Gene expression was normalized to the housekeeping gene *36B4 (encodes acidic ribosomal phosphoprotein P0)* or *glyceraldehyde 3‐phosphate dehydrogenase* in the case of MSC and ribosomal protein L30 (*RPL30*) in case of HUVECs according to the formula 2^(Ct housekeeping– Ct gene of interest)^.

### 2.9 Ki‐67 cell proliferation assay

MSC (5000 cells/cm^2^) or HUVECs (10,000 cells/cm^2^) were seeded in 48 wells plates on plastic or containing ECM of previously prepared Day 11 osteogenically differentiated MSC cultures (20% or 2% O_2_ ± GO‐201). Cells were incubated for 3 days under normoxia. After incubation, cells were trypsinized, pelleted down, fixed with dropwise addition of 70% ethanol, and placed on ice for 5 min followed by freezing at −20°C for 30 min. Next, cells were pelleted down, washed twice with 1 ml PBS + 1% bovine serum albumin. After pelleting down the cells, staining solution (55 µl PBS/5% FBS + 1.0 µl mouse anti‐Ki‐67 antibody conjugated with Alexa 488; Thermo Fisher Scientific) was added and incubated for 30 min in the dark at room temperature. After incubation, cells were washed twice with PBS containing 5% FBS and resuspended in nuclear stain solution (55 µl PBS/5% FBS + 0.5 µl propidium iodide). Cells were sorted using the Accuri C6 flow cytometer and analyzed with the associated software (BD Biosciences).

### 2.10 Cell metabolic activity assay

The metabolic activity assay was performed as previously reported (Pierri et al., 2012). Briefly, MSC (5000 cells/cm^2^) were seeded on previously generated ECMs of MSC cultured under 20% or 2% O_2_ ± GO‐201 in 48‐well plates and incubated for 6, 24, or 48 h at 37°C/5% CO_2_. After incubation, cell metabolic activity was assessed using PrestoBlue™ reagent (Invitrogen) according to the manufacturer's specifications and absorbance was measured between 560 nm (excitation) and 590 nm (emission), using a Wallac Victor 1420 (GMI).

### 2.11 Statistical analysis

The data are shown as representative of multiple independent experiments. Quantitative graphs are shown as mean ± *SD*. One‐way analysis of variance was performed, followed by the Bonferroni post hoc test to calculate statistical significance unless specified otherwise. A *p* value ≤0.05 was considered significantly different.

## 3 RESULTS

### 3.1 Hypoxia stabilizes HIF1α and upregulates MUC1 and *VEGFA* in human osteoblasts

**Figure 1:**
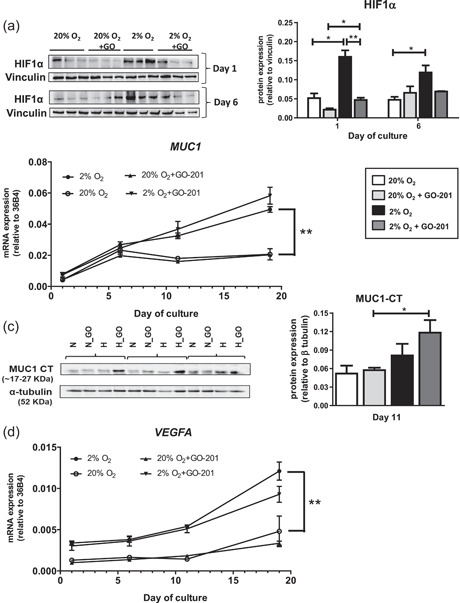
Effect of hypoxia and MUC1‐CT inhibition on HIF1α, MUC1, and *VEGF* expression. (a) HIF1α protein expression on Days 1 and 6 of osteogenic differentiation of human MSC under 20% or 2% oxygen with or without GO‐201. (b) Gene expression of *MUC1* on the Days 1, 6, 11, and 19. (c) Protein expression of MUC1 on Day 11. (d) Gene expression of *VEGF* on Days 1, 6, 11, and 19. Ribosomal phosphoprotein 36B4 was used as a housekeeping gene and vinculin or α‐tubulin was used to correct for protein expression. Results are representative for multiple independent experiments (*p < 0.05; **p < 0.005; ***p < 0.001). Bars represent averages ±* SD*. HIF1α, hypoxia‐inducible factors 1α; MUC1, Mucin 1; VEGF, vascular endothelial growth factor

To assess the effect of hypoxia, we measured HIF1α on Days 1 and 6 and found 3‐ and 2.5‐fold higher protein levels, respectively, in hypoxic cells compared to cells under normoxic conditions. In addition, MUC1‐CT inhibition under 2% oxygen reduced HIF1α levels on Day 1 compared to untreated cells (Figure 1a). Furthermore, we explored the effect of hypoxia on MUC1 expression and found that hypoxia significantly increased *MUC1* during osteogenic differentiation of MSCs (Figure 1b). This was confirmed by increased MUC1‐CT protein expression at Day 11 (Figure 1c). Hypoxia also increased *VEGF* expression during the course of MSC differentiation towards osteoblasts, which is independent of MUC1‐CT inhibition (Figure 1d). In addition, *CTGF* expression was increased in hypoxic osteoblast cultures on Day 11 but there was no effect on *PDGF‐A* and *PDGF‐B* expression (Figure S1).

### 3.2 Hypoxia and/or MUC1‐CT inhibition diminish matrix mineralization by human osteoblasts

**Figure 2:**
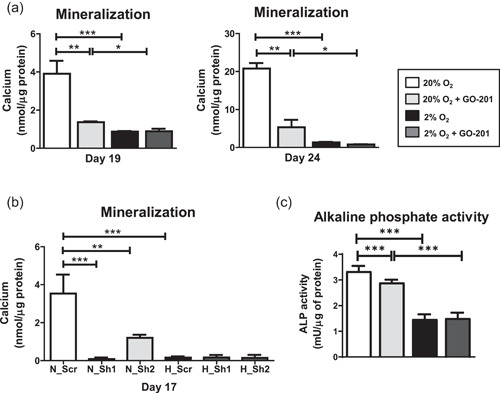
Effect of hypoxia and MUC1‐CT inhibition on osteogenic differentiation of MSCs. (a) Mineralization of extracellular matrix (ECM) on Days 19 and 24 of osteogenic differentiation of MSC under 20% or 2% oxygen with or without GO‐201. (b) Mineralization of ECM on Day 17 of osteogenic differentiation of MSC under 20% or 2% oxygen with scrambled (Scr) or short hairpin RNAs against MUC1 (Sh1 and Sh2). (c) Alkaline phosphatase (ALP) activity measured in cell lysates on Day 11 of osteogenic differentiation of human MSC under 20% or 2% oxygen with or without GO‐201 (*p < 0.05; ***p < 0.001). Bars represent averages ±* SD*. MSC, mesenchymal stromal cell; MUC1, Mucin 1

Next, we compared the effect of hypoxia and normoxia on mineralization of osteoblast matrices with or without MUC1‐CT inhibition on Days 19 and 24 of osteogenic differentiation. Mineralization was reduced under hypoxia compared to normoxia by 4.5‐fold and 16‐fold on Days 19 and 24, respectively (Figure 2a). Similarly, MUC1‐CT inhibition also reduced mineralization of osteoblasts matrix under normoxia 2.9‐ and 5.2‐fold on Days 19 and 24, respectively. Under hypoxia, GO‐201 did not modulate mineralization of osteoblasts (Figure 2a). To validate these findings, we used two individual short hairpin RNAs designed against *MUC1* and measured mineralization of osteoblast matrix on Day 17. *MUC1* silencing efficiency of the shRNAs was measured by RT PCR, which was above 80% (Figure S2). We found that hypoxia or *MUC1* interference under normoxia reduced osteoblast mineralization (Figure 2b). Similar to MUC1‐CT inhibition, MUC1 shRNA under hypoxia did not modulate osteoblast mineralization. ALP is an osteoblast differentiation marker and when we measured its activity, we found that it was significantly reduced under hypoxia as well as following MUC1‐CT inhibition (Figure 2c). In line with the mineralization data, there was no further decrease in ALP activity following MUC1‐CT inhibition under hypoxia. Taken together, these results showed that either hypoxia or MUC1‐CT inhibition reduced matrix mineralization of human osteoblasts.

### 3.3 Hypoxia and/or MUC1‐CT inhibition alters the proteomic landscape of ECM produced by osteoblasts

**Figure 3:**
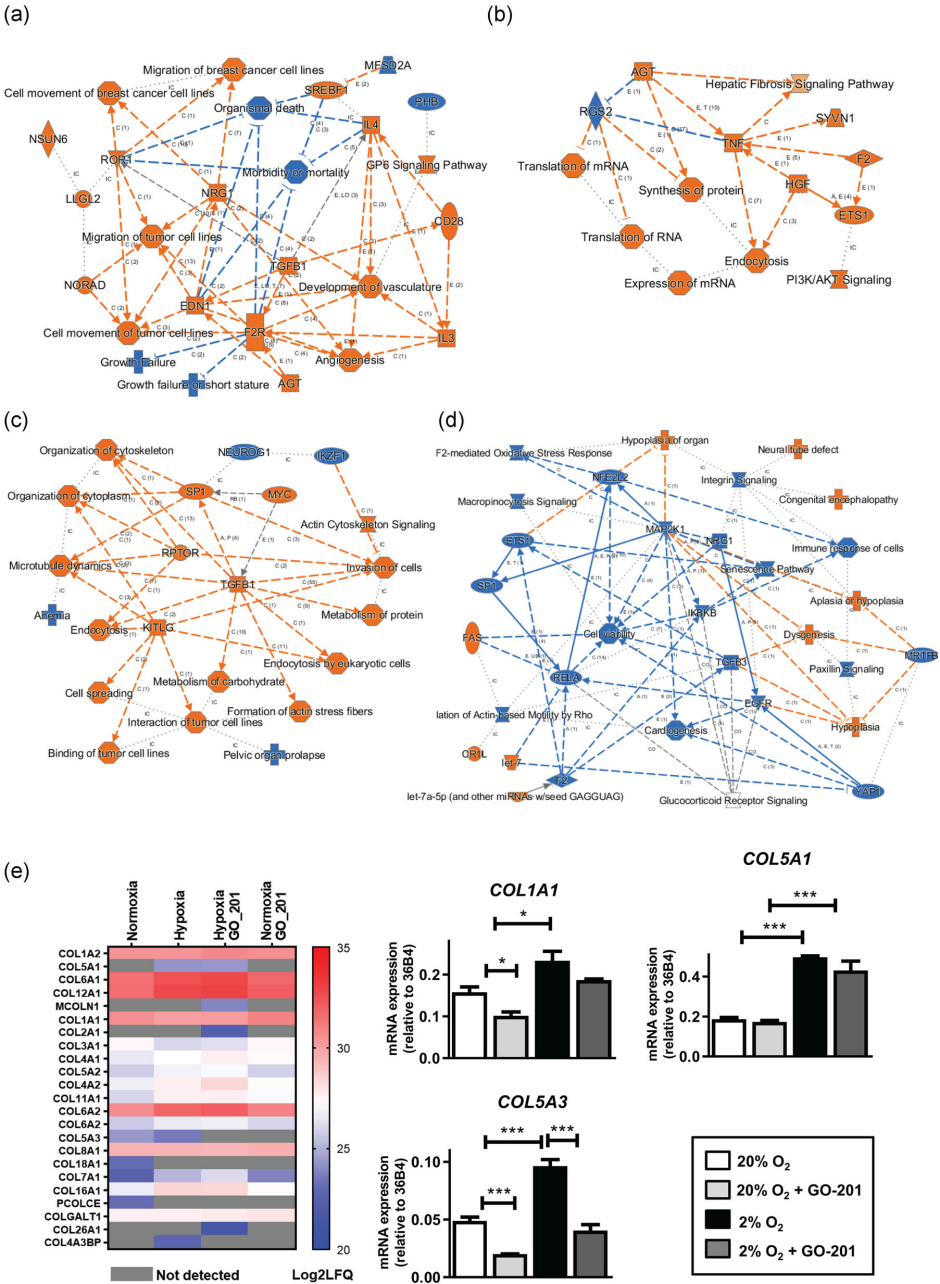
Effect of hypoxia and MUC1‐CT inhibition on the proteomic landscape of ECM of osteogenic differentiation of MSCs. (a) Pathway enrichment analysis of proteins detected in hypoxic ECM versus normoxic ECM; proteins either uniquely present or up‐or downregulated (≥2‐fold) between the two groups were included for the analysis. Activated pathways are shown in orange and deactivated pathways are shown in blue. (b) Pathway enrichment analysis of proteins detected in normoxic‐GO‐201 ECM versus normoxic ECM; proteins either uniquely present or up‐or downregulated (≥2‐fold) between the two groups were included for the analysis. Activated pathways are shown in orange and deactivated pathways are shown in blue. (c) Pathway enrichment analysis of proteins detected in hypoxic‐GO‐201 ECM versus hypoxic ECM; proteins either uniquely present or up‐or downregulated (≥2‐fold) between the two groups were included for the analysis. Activated pathways are shown in orange and deactivated pathways are shown in blue. (d) Pathway enrichment analysis of proteins detected in hypoxic‐GO‐201 ECM versus normoxic‐GO‐201 ECM; proteins either uniquely present or up‐or downregulated (≥2‐fold) between the two groups were included for the analysis. Activated pathways are shown in orange and deactivated pathways are shown in blue. (e) Heat map representing the abundance (LFQ values) of all the collagen proteins detected in the four groups of ECM samples combined. Red: high abundance, blue: low abundance, grey: not detected. (f) Gene expression of *COL1A1, COL5A1*, and *COL5A3* on Day 11 of osteogenic differentiation of human MSC under 20% or 2% oxygen with or without GO‐201. Ribosomal phosphoprotein 36B4 was used as a housekeeping gene (*p < 0.05; **p < 0.005; ***p < 0.001). Bars represent averages ± *SD*. ECM, extracellular matrix; MSC, mesenchymal stromal cell; MUC1, Mucin 1

We compared the proteomic composition of ECMs produced by normoxic or hypoxic osteoblasts at Day 11 in the presence or absence of GO‐201. MS yielded protein lists based on their representing peptide sequences. Proteins represented by at least two peptides were selected for the analysis. Among the samples, 3,182 different proteins were detected (containing ≥2 peptides). The number of uniquely expressed proteins in the ECMs under hypoxia and normoxia was 390 and 285, respectively, whereas 1840 proteins were shared between these groups. In addition, compared to normoxia, seventy proteins were upregulated and 25 proteins were downregulated twofold or more under hypoxia (Figure S3a). Ontological analysis, as assessed by Ingenuity pathway analysis, of up‐and downregulated and uniquely expressed proteins when comparing hypoxic ECM with normoxic ECM showed that the overrepresented pathways due to hypoxia included “Angiogenesis and vascular development”, “Cell migration”, and “GP6 signaling”. The underrepresented pathways included “Growth failure” and “Mortality” (Figure 3a).

MUC1‐CT inhibition in normoxic osteoblasts upregulated 31 proteins (≥2 fold) compared to control while 338 proteins were uniquely expressed. In the same conditions, MUC1‐CT inhibition downregulated 30 proteins (≥2 fold) compared to control while 255 proteins were uniquely expressed (Figure S3b). Ontological analysis of up‐and downregulated and uniquely expressed proteins when comparing normoxic ECM with normoxic+GO‐201 ECM revealed the overrepresented pathways “Protein synthesis”, “mRNA translations”, “Endocytosis”, and “PI3K/AKT signaling”. The underrepresented pathways included “RGS2” only (Figure 3b).

In the ECM of MUC1‐CT inhibited hypoxic osteoblasts, 298 proteins were uniquely expressed following GO‐201 treatment, while 268 proteins were uniquely expressed in the hypoxic control condition. In total, 12 proteins were upregulated and 11 proteins downregulated under GO‐201 treatment by twofold or more compared to control (Figure S3c). Ontological analysis of up‐and downregulated and uniquely expressed proteins when comparing hypoxic ECM with hypoxic+GO‐201 ECM revealed that overrepresented pathways were “Organization of cytoskeleton”, “Actin cytoskeleton signaling”, “TGFβ1”, and “Invasion of cells” and “Metabolism of carbohydrates”. The underrepresented pathways included “DNA damage recognition in GG‐NER”, and “Clathrin‐mediated endocytosis” (Figure 3c).

As MUC1‐CT inhibition and hypoxia yielded a similar functional effect during osteoblast differentiation, we compared the proteomic profiles of ECMs generated from osteoblasts from these two conditions. We found that 317 proteins were uniquely expressed under normoxia following GO‐201 treatment, while 338 proteins were uniquely expressed in the hypoxic control condition. In total, 24 proteins were upregulated and 59 proteins downregulated under normoxic_GO‐201 treatment by twofold or more compared to hypoxia control (Figure S3d). Ontological analysis of uniquely expressed and upregulated proteins under normoxia with GO‐201 treatment versus the hypoxic control showed that pathways overrepresented were “Hypoplasia”, “Dysgenesis”, “FAS”, and “let‐7 signaling”. The underrepresented pathways included “Cell viability”, “Senescence pathway”, and “Integrin signaling” (Figure 3d).

We noticed that the collagen family is one of the major groups of proteins affected under hypoxia and/or MUC1‐CT inhibition as shown in the heat map (Figure 3e). We confirmed this by showing that hypoxia significantly stimulated the expression of *COL1A1, COL5A1*, and *COL5A3* at Day 11 of osteoblast cultures (Figure 3f). MUC1‐CT inhibition downregulated *COL1A1* under normoxia but not under hypoxia. MUC1‐CT inhibition also downregulated *COL5A3*, both under normoxia and hypoxia (Figure 3f).

### 3.4 MUC1‐CT inhibition affects phospho‐AMPK/mTORC1/phospho‐S6 pathway in MSCs

**Figure 4:**
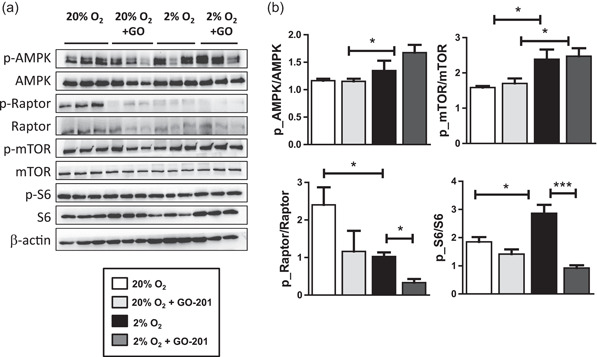
Effect of hypoxia and MUC1‐CT inhibition on AMPK/mTORC1/S6K pathway in osteogenic differentiation of MSCs. (a) Immunoblots of protein expression on Day 1 of osteogenic differentiation of MSC cultured under 20% or 2% oxygen with or without GO‐201. (b) Quantification of phosphorylated over total protein ratio's relative to β‐actin (*p < 0.05). Bars represent averages ± *SD*. AMPK, AMP‐activated protein kinase; MSC, mesenchymal stromal cell; MUC1, Mucin 1

We looked at the expression and phosphorylation of several proteins involved in the AMPK/mTORC1/S6 pathway as it is involved in the osteogenic differentiation of MSC (Pantovic et al., 2013). Here, we observed that MUC1‐CT inhibition under hypoxia significantly increased phosphorylation of AMPK after 24 h of treatment compared to normoxic cells that were treated with GO‐201 but not compared to hypoxia alone (Figure 4a,b). In addition, compared to normoxia and independent of GO‐201, hypoxia significantly increased phosphorylation of mTOR while phosphorylation of raptor was decreased. GO‐201 reduced hypoxia‐induced phosphorylation of S6 (Figure 4a,b). MUC1‐CT inhibition under normoxia led to a decreased phosphorylation of AMPK but no other effects were seen when MUC1 was inhibited at this time point (Figure 4a,b).

### 3.5 Collagenous changes in the ECM following hypoxia and/or MUC1‐CT inhibition are mediated through activation of TGFβ‐smad and NFκB pathways

**Figure 5:**
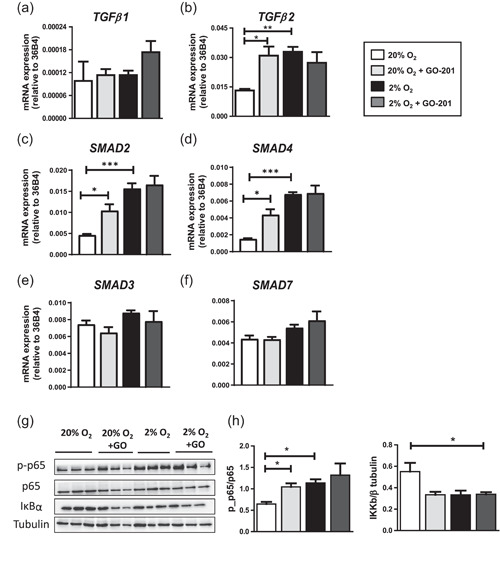
Effect of hypoxia and MUC1‐CT inhibition on IKK/NFκB pathway in osteogenic differentiation of MSCs. (a, b) Gene expression of *TGFβ1* (left panel) and *TGFβ2* (right panel). (c–f) Gene expression of SMAD2, SMAD4, SMAD3, and SMAD7. Ribosomal phosphoprotein 36B4 was used as a housekeeping gene. (g) Immunoblots of protein expression on Day 1 (left panels) and Day 6 (right panels) of osteogenic differentiation of MSC cultured under 20% or 2% oxygen with or without GO‐201. (h) Quantification of proteins relative to *β*‐tubulin (*p < 0.05; **p < 0.005; ***p < 0.001). Bars represent averages ± *SD*. MSC, mesenchymal stromal cell; MUC1, Mucin 1; NFκB, nuclear factor‐κB; TGFβ, transforming growth factor‐beta

As hypoxia promotes collagen formation in dermal fibroblasts by TGF‐β1/Smad signaling (Mingyuan et al., 2018), we investigated this in human osteoblasts. We found that hypoxia and/or MUC1‐CT inhibition did not change *TGFβ1* (Figure 5a). However, *TGFβ2* was 2.5‐fold upregulated under hypoxia and 2.4‐fold following MUC1‐CT inhibition at Day 11 of culture (Figure 5b). Downstream of TGF*β*, hypoxia or MUC1‐CT inhibition under normoxia upregulated *SMAD2* and *SMAD4* (Figure 5c,d) but there was no change in inhibitory SMADs (*SMAD3* and *SMAD7*) under hypoxia and/or MUC1‐CT inhibition (Figure 5e,f).

As the inhibition of IKK/nuclear factor‐κB (NF‐κB) pathway enhances bone matrix formation in vitro and in vivo (Chang et al., 2013, 2009a), while hypoxia or TGFβ signaling is shown to activate NF‐κB signaling (Oliver et al., 2009), we studied this pathway in MSC‐derived osteoblasts. Here we report that hypoxia and MUC1‐CT inhibition both significantly decreased total IκBα level after 24 h of treatment, although phosphorylation of IκBα was not detected (Figure 5g,h). Following degradation of IkK, phosphorylation of NFκB p65 significantly increased under hypoxia and following MUC1‐CT inhibition after 24 h of treatment (Figure 5g,h).

### 3.6 ECMs of hypoxic and MUC1‐CT inhibited osteoblasts have opposite effects on osteogenesis

**Figure 6:**
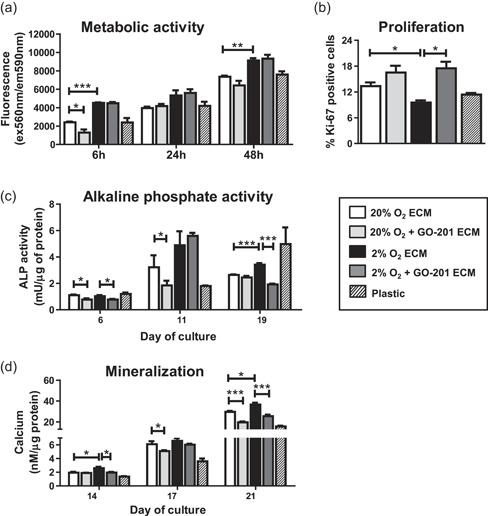
Effect of ECMs generated from normoxic or hypoxic osteoblasts with or without GO‐201 on osteogenic differentiation of MSC. (a) Metabolic activity of MSC cultured on plastic or different ECMs for 6, 24, and 48 h. (b) Percentage of Ki‐67 positive MSC cultured for 3 days over plastic or different ECMs counted by flow cytometry. (c) Alkaline phosphatase (ALP) activity of osteogenically differentiating MSC on Days 6, 11, and 19 cultured on plastic or different ECMs. (d) Mineralization of extracellular matrix (ECM) of osteogenically differentiating MSC on Days 14, 17, and 21 cultured on plastic or different ECMs (*p < 0.05; **p < 0.005; ***p < 0.001). Bars represent averages ± *SD*. ECM, extracellular matrix; MSC, mesenchymal stromal cell

Next, we studied the effects of different ECMs on MSC and their osteogenic differentiation, in line with our previous work showing that alterations in ECMs have consequences for osteogenesis (Baroncelli et al., 2018). We observed increased metabolic activity of MSC cultured for 6 h or 48 h on ECMs of hypoxic osteoblasts (Figure 6a). Metabolic activity of MSC was reduced when cultured on ECM of MUC1‐CT inhibited normoxic osteoblasts for 6 h (*p* < 0.05) but was unaffected later on. However, ECMs of MUC1‐CT‐inhibited hypoxic osteoblasts did not affect the metabolic activity of MSC compared to hypoxic control ECMs. Although not significant, hypoxic ECMs consistently led to the higher metabolic activity of MSC, irrespective of MUC1‐CT inhibition (Figure 6a). We also checked for the proliferation of MSC cultured on different ECMs, using Ki‐67 as a proliferation marker. We found that ECMs of hypoxic osteoblasts inhibited the proliferation of MSC compared to those cultured on ECMs of normoxic osteoblasts, which was reversed when cultured on ECMs of MUC1‐CT‐inhibited hypoxic osteoblasts (Figure 6b). Finally, we assessed the osteogenic potential of these ECMs by measuring ALP activity and matrix mineralization of osteoblasts cultured on top of these ECMs. Compared to plastic, the ECMs accelerated osteoblast differentiation as seen by the increased ALP activities at Day 6 and a delayed increase at Day 11 for the plastic‐seeded cultures (Figure 6c). We observed a significant reduction in ALP activity of osteoblasts cultured on ECM of MUC1‐CT‐inhibited hypoxic or normoxic osteoblasts on Day 6 compared to the ECMs of untreated MSC. The effect was persistent at Day 11 in ECM of MUC1‐CT‐inhibited normoxic osteoblasts (Figure 6c). In a similar experimental setup, we measured deposited calcium as an indicator of matrix mineralization by osteoblasts. All 4 ECMs were osteopromotive compared to plastic (Figure 6d). More specifically, ECMs of hypoxic osteoblasts significantly increased the mineralization of osteogenically differentiating MSC on Days 14 and 21 compared to normoxic ECMs. Moreover, the ECMs of both MUC1‐CT‐inhibited normoxic and hypoxic osteoblasts significantly affected the mineralization of osteogenically differentiating MSC on Day 21 (Figure 6d). Overall, these results suggest that ECMs of hypoxic osteoblasts enhance metabolic activity of MSC, reduce their proliferation and accelerate their osteogenic differentiation, whereas MUC1‐CT‐inhibited ECMs counteracted these effects.

### 3.7 MUC1‐CT inhibition reduced angiogenic potential of ECM independent of oxygen concentration

**Figure 7:**
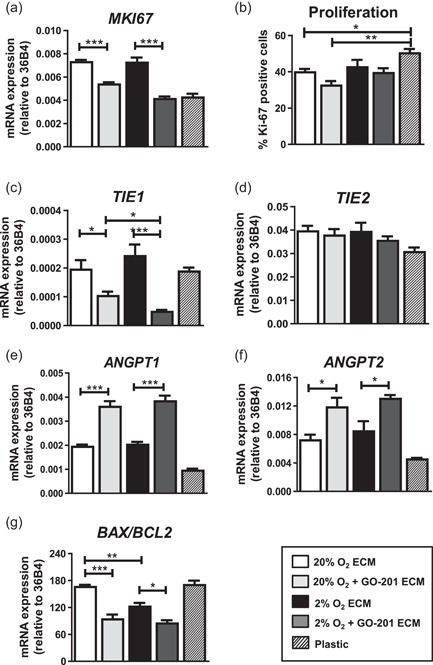
Effect of ECMs generated from normoxic or hypoxic osteoblasts with or without GO‐201 on the behavior of HUVECs. (a) Gene expression of *Ki‐67* in HUVECs cultured for 5 days on plastic or ECMs generated from osteogenically differentiated human MSC under 20% or 2% oxygen with or without GO‐201. (b) Percentage of Ki‐67 positive HUVECs cultured for 3 days over plastic or different ECMs counted by flow cytometry. (c–f) Gene expression at Day 3 of *TIE‐1, TIE‐2, ANG‐1*, and *ANG‐2*. (g) The ratio of mRNA expression of *BAX/BCL2*. Ribosomal phosphoprotein 36B4was used as housekeeping gene (*p < 0.05; **p < 0.005; ***p < 0.001). Bars represent averages ±* SD*. ECM, extracellular matrix; HUVEC, human umbilical vein endothelial cell; mRNA, messenger RNA

To investigate the effects of different ECMs on angiogenesis, we cultured HUVECs on top of the ECMs prepared from human osteoblasts cultured under normoxia or hypoxia with and without GO‐201. We assessed the proliferation of HUVECs on ECMs by sorting Ki‐67 positive cells after 3 days of culture and MKI67 mRNA (encoding Ki‐67) expression on Day 5 of culture. ECMs derived from MUC1‐CT‐inhibited normoxic or hypoxic osteoblasts significantly reduced the expression of *MKI67* to the level of cells cultured on plastic (Figure 7a). Although, the percentage of Ki‐67 positive cells did not change among the four ECM conditions. The normoxic ECMs inhibited cell proliferation versus osteoblasts cultured on plastic (Figure 7b). Formation of new blood vessels (sprouting) involves three sequential cells types, that is, pharynx cells, stalk cells, and tip cells (leading cell) (Gerhardt, 2013), the latter of which are characterized by an increased ratio of TIE1/TIE2 (Savant et al., 2015). We observed that ECMs of MUC1‐CT‐inhibited normoxic or hypoxic osteoblasts reduced *TIE1* expression (Figure 7c), whereas *TIE2* was unaffected (Figure 7d). We also checked for expression of angiopoietins since angiopoietin 1 (ANGPT1) along with TIE2 promotes vascular stabilization and maturation and therefore reduces “tip‐ness” of endothelial cells (Brindle et al., 2006). We found significantly increased expression of *ANGPT1* and ANGPT2 by ECMs of MUC1‐CT‐inhibited normoxic or hypoxic osteoblasts (Figure 7e,f). In addition, ECMs of hypoxic or MUC1‐CT‐inhibited osteoblasts led to reduced apoptosis in HUVECs as determined by the ratio of *BAX* to *BCL2* expression (Figure 7g). Altogether, ECMs of MUC1‐CT‐inhibited osteoblasts reduced HUVEC proliferation, apoptosis, and formation of tip cells and increased vessel maturation, while ECMs of hypoxic osteoblasts reduced apoptosis in HUVECs without affecting the cell proliferation and tip cell markers.

## 4 DISCUSSION

**Figure 8:**
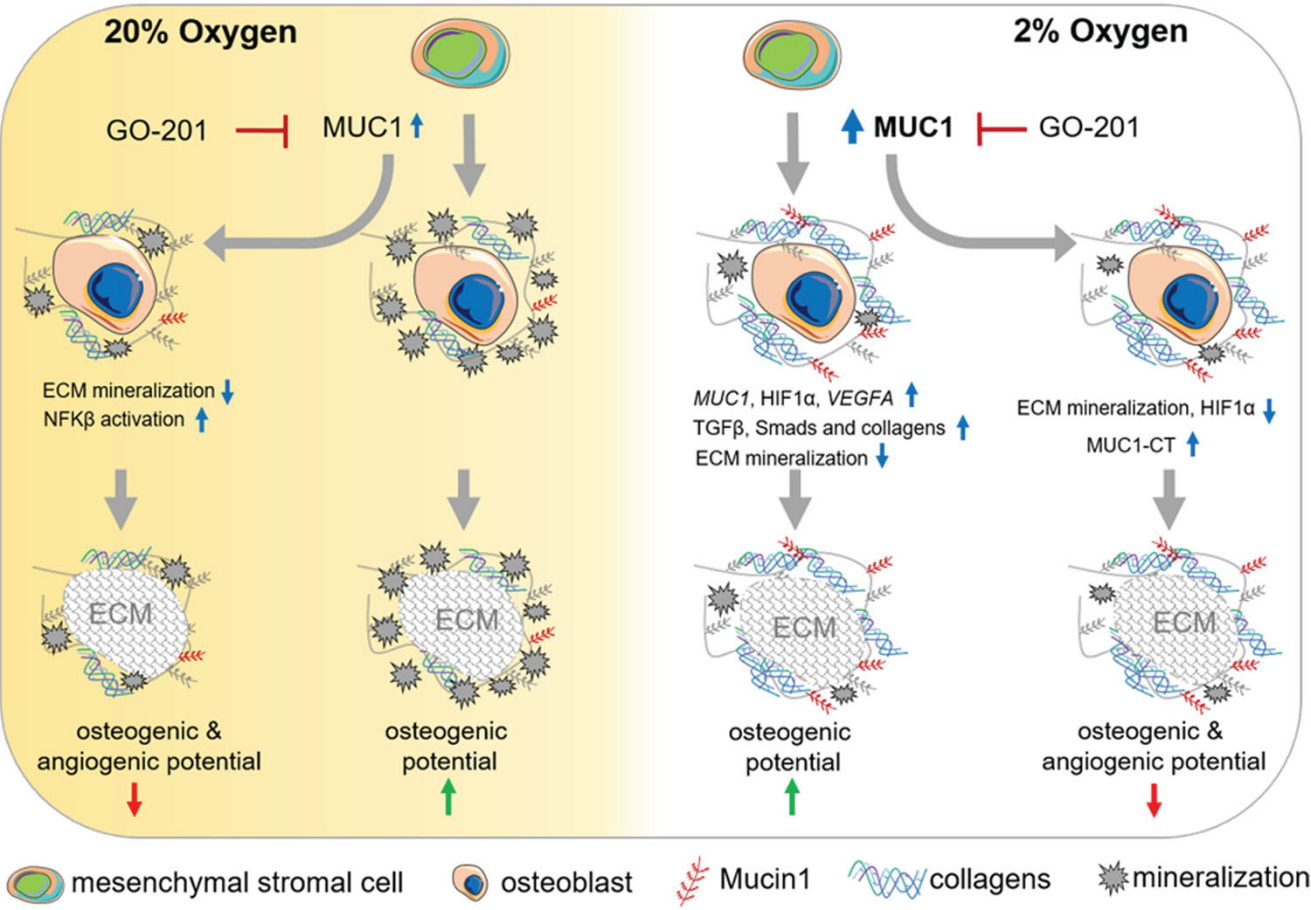
Schematic model of hypoxia‐induced Mucin 1 regulating the osteogenic differentiation of human mesenchymal stromal cells and properties of their extracellular matrix. *MUC1* is moderately expressed in osteoblasts under 20% oxygen (normoxia) (blue arrow in the yellow panel). Inhibition of *MUC1* by GO‐201 (red symbol) under normoxic conditions reduced ECM mineralization, activated NFkB pathway, reduced osteogenic, and angiogenic potentials of ECMs in osteoblasts (in the left yellow panel). Hypoxia upregulated *MUC1* and reduced mineralization of ECM in osteoblasts (blue arrow in the white panel). Hypoxic ECM had higher osteogenic potential compared to normoxic ECM that was reversed by MUC1 inhibition under hypoxia (white panel). ECM, extracellular matrix; MUC1, Mucin 1; NFκB, nuclear factor‐κB

In this study, we explored the effects of hypoxia and/or MUC1 inhibition on osteogenic differentiation of human MSCs and their ECM. We claim that hypoxia upregulates MUC1 in osteoblasts. Hypoxia or MUC1‐CT inhibition delays the osteogenic differentiation of MSCs, alters the proteomic composition of their ECMs, and leads to differential effects on subsequent osteogenesis and angiogenesis (Figure 8).

We found that hypoxia reduced osteogenic differentiation and ECM mineralization of MSCs, which corroborated with findings of Utting et al. (2006) in rat osteoblasts. In contrast to our results, Yu et al. (2019) reported that cellular hypoxia promotes osteogenic differentiation of MSCs. However, Yu et al. (2019) had simulated the effect of hypoxia by cobalt chloride (CoCl_2_) unlike Utting et al. (2006) and our study. These contradictory findings raise concerns about the use of CoCl_2_ to induce hypoxic effects, as it may stabilize HIFs and other proteins to an extent beyond the physiological stabilization by hypoxia and thereby produce differences in osteogenic endpoints. In a mouse model, the lack of HIF1α in osteoblasts reduced bone volume (Shomento et al., 2010). On the basis of our findings that hypoxia upregulated COL1A1, COL5A1, and COL5A3 in osteoblasts among which, collagen type I contributes to approximately 90% of the protein‐part of bone matrix, this may indicate that HIF1α helps to maintain bone volume by increasing collagen production.

MUC1 is upregulated by hypoxia and inhibition of its signaling mimics the effect of hypoxia on differentiation of osteoblasts and ECM mineralization. Inhibition of MUC1‐CT destabilized HIF1α indicating that MUC1 might interfere in the hydroxylation or proteasomal degradation of HIF1α (Chaika et al., 2012). This also suggests that the C‐terminal part of MUC1 could be a key transcription factor to control the “stemness” of hMSCs in the hypoxic niche of bone marrow as shown in colorectal cancer (Li et al., 2020).

Hypoxia activates AMPK signaling (Dengler, 2020). We noticed changes in the AMPK/mTORC1/S6K pathway as an early wave during osteogenic differentiation. We found that that hypoxia alone did not change the phosphorylation of AMPK. However, in combination with MUC1‐CT inhibition phosphorylation of AMPK at threonine (position 172) was activated. We also confirmed that hypoxia activated the mTORC1/S6K pathway, which may not have been directly regulated by AMPK alone. Similarly, MUC1‐CT inhibition along with hypoxia decreased the mTORC1/S6K pathway but had no effect under normoxia. This suggests that MUC1‐CT under hypoxia activates the AMPK/mTORC1/S6K pathway during early osteogenic differentiation of MSC. However, these findings do not exclude the possibility of other pathways involved in this process. We and several other studies found that hypoxia upregulates growth factors such as *VEGF* in osteoblasts (Chen et al., 2012). However, the consistent upregulation of *VEGF* during osteogenic differentiation of MSCs under hypoxia indicates that it might not be regulated by the AMPK/mTORC1 pathway.

The TGF‐β/SMAD signaling pathway is one of the prominent pathways regulating ECM formation. In canonical TGF*β* signaling, TGF*β* binds and activates the TGF*β*Rs leading to the activation of the TGF‐β/SMAD signaling pathway. In our study, we found upregulation of *TGFβ2* expression under hypoxia and/or MUC1‐CT inhibition in osteogenically differentiating MSCs, which correlates with the upregulation of *SMAD2* and *SMAD4* but not *SMAD3* and *SMAD7* (inhibitory SMADs). This suggests that the hypoxia‐MUC1 pathway activates SMAD2/4 via TGFβ2 but not TGFβ1 in osteogenically differentiating MSCs and thereby remodeling the ECM. Besides SMAD activation, TGFβ signaling also activates NFkB that is negatively associated with osteoblast differentiation and ECM formation (Chang et al. 2009a). In addition, MUC1‐CT is reported to activate NFkB signaling by interacting and causing proteasomal degradation of IκBs (Ahmad et al., 2007). We found reduced levels of IκBα under the influence of MUC1‐CT inhibition and/or hypoxia that could be due to its proteasomal degradation. Following the lower level of IκBα, as expected, phosphorylation of the p65 subunit of NFkB complex was increased under MUC1‐CT inhibition and/or initial exposure of hypoxia. Phosphorylation of p65 negatively regulates the *COL1A1* gene in human fibroblasts (Beauchef et al., 2012). We found a similar correlation of p65 phosphorylation in human osteoblasts where inhibition of MUC1‐CT increased p65 phosphorylation might lead to decreased expression of *COL1A1*, although we did not provide direct evidence for this.

Previous studies from our group have successfully applied devitalized ECM to study in vitro and in vivo osteogenic differentiation and bone formation (Baroncelli et al., 2018). Baroncelli et al. (2018) reported that in vitro cell‐deposited ECM is osteopromotive and shared more than 50% homology with human bone proteome. In this study, we have identified the proteins in the ECM of normoxic or hypoxic osteoblasts with or without MUC1‐CT inhibition. Hypoxia upregulated proteins are involved primarily in angiogenesis, cell migration, and TGFβ1 signaling. Surprisingly, hypoxic ECM shared similarities of pathways with the MUC1‐CT inhibited normoxic ECM particularly in up‐ regulated proteins. Collagens were mostly upregulated in hypoxic ECM, which were in contrast with the fact that they are considered as an indicator of osteogenesis (Tsai et al., 2010). However, mineralization of the matrix involves cross‐linking of collagens (Knott & Bailey, 1998). Although the collagens were upregulated, whether the reduction in matrix mineralization under hypoxia and/or MUC1‐CT inhibition resulted from lack of cross‐linking among collagens, requires further investigation. Alternatively, massive upregulation of collagens may disturb the natural organic ECM composition, which may hamper normal ECM formation and mineral crystal incorporation. Cell‐derived ECM obtained from an MSC‐HUVEC coculture (Carvalho et al., 2019) or in combination with hydroxyapatite (Tour et al., 2011) displayed higher cell anchoring properties and enhanced osteogenic properties of ECM. Similarly, Somaiah et al. (2015) have shown that collagen type 1 coating increased MSCs attachment, survival, and their osteogenic differentiation. In our study, hypoxia increased the expression of collagens and reduced mineralization, which may have enhanced anchoring properties within ECM and thereby facilitated MSCs to attach and differentiate towards osteoblasts. We identified *MUC1* as a hypoxia‐sensitive gene; however, the ECMs of MUC1‐inhibited osteoblasts reduced osteogenic differentiation of MSCs under both hypoxia and normoxia. This could be due to the basal expression of *MUC1* under normoxia and/or its cross‐linking with other pathways that require further investigations.

Bone growth and repair require osteogenesis and angiogenesis to occur simultaneously, and the same microenvironment should enable both osteoblasts and endothelial cells to function well. Therefore, we also investigated angiogenesis on the devitalized ECMs. Collagens in general, act as proangiogenic stimuli in bone matrix (Neve et al., 2014). Our data showed increased collagen expression under hypoxia along with enriched angiogenic pathways in comparative proteomic analyses of ECMs from normoxic osteoblasts and hypoxic osteoblasts, suggesting that ECM of hypoxic osteoblasts have increased angiogenic potential. Hypoxia itself is a proangiogenic stimulus in tissue but it is noteworthy that ECM of osteoblasts differentiated under hypoxia also holds proangiogenic cues without having any metabolic activity. Similar to osteogenic markers, inhibition of MUC1 reduced the angiogenic potential of ECMs in both normoxic and hypoxic osteoblasts. Notably, *TIE1*, a proangiogenic marker, was significantly suppressed in the HUVECs cultured on ECMs of MUC1‐inhibited hypoxic osteoblasts compared to ECM of MUC1‐inhibited normoxic osteoblasts, which could be correlated with increased expression of *MUC1* in hypoxic osteoblasts.

In conclusion, our study claims that MUC1 is critical for hypoxia‐mediated changes during osteoblastogenesis that not only alters the proteomic landscape of the ECM but thereby also modulate its osteogenic and angiogenic potentials. The study emphasizes that it is pivotal to understand the role of hypoxia and MUC1 in developing customized ECM scaffolds to induce osteogenesis.

## CONFLICT OF INTERESTS

The authors declare that there are no conflict of interests.

## AUTHOR CONTRIBUTIONS

**Pavitra K. Jadaun, Marijke Koedam, and Bram C. van der Eerden**: designed the experiments. **Pavitra K. Jadaun, Marijke Koedam, and Shuang Zhang**: performed the experiments and analyzed the data. **Jeroen Demmers**: provided the LC‐MS/MS facility and data analysis. **Pavitra K. Jadaun and Bram C. van der Eerden**: wrote the manuscript. **Johannes P. van Leeuwen, Jeroen Demmers, and Suvro Chatterjee**: helped in revising the manuscript. All the authors reviewed and approved the manuscript.

## Supporting information

Supporting information.

## Data Availability

The datasets generated during and/or analyzed during the current study are available from the corresponding author on reasonable request.
