# Supplementary material for: Inhibition of hypoxia‐induced Mucin 1 alters the proteomic composition of human osteoblast‐produced extracellular matrix, leading to reduced osteogenic and angiogenic potential
Source: J Cell Physiol. 2021 Oct 22;237(2):1440–54. doi: 10.1002/jcp.30617 (PMC9298310; doi:10.1002/jcp.30617)
Supplement: Supplementary file 1 — Supporting information. [file JCP-237-1440-s001.docx]

**Inhibition of hypoxia-induced Mucin 1 alters proteomic composition of human osteoblast-produced extracellular matrix, leading to reduced osteogenic and angiogenic potential**

**Running title:**

**Concerted actions of hypoxia and Mucin 1 on human osteoblastogenesis**

Pavitra K. Jadaun^1,2^, Shuang Zhang^1^, Marijke Koedam^1^, Jeroen Demmers^3^, Suvro Chatterjee^2^, Johannes P. van Leeuwen^1^, and Bram C. van der Eerden^1*^

^1^Laboratory for Calcium and Bone Metabolism, Department of Internal Medicine, Erasmus University Medical Center, Rotterdam, the Netherlands

^2^Vascular Biology Laboratory, AU-KBC Research Centre, Anna University, Chennai, India

^3^Proteomics Centre, Erasmus University Medical Center, Rotterdam, the Netherlands

***^*^Corresponding author:***

Bram C.J. van der Eerden, PhD

Laboratory for Calcium and Bone Metabolism

Department of Internal Medicine, room Ee585b

Erasmus University Medical Center

Dr Molewaterplein 40

3015 GD Rotterdam

The Netherlands

Tel: +31-10-7032841

Fax: +31-10-7044862

Email: b.vandereerden@erasmusmc.nl

**Supplementary materials**

**Supplementary Figures**

**
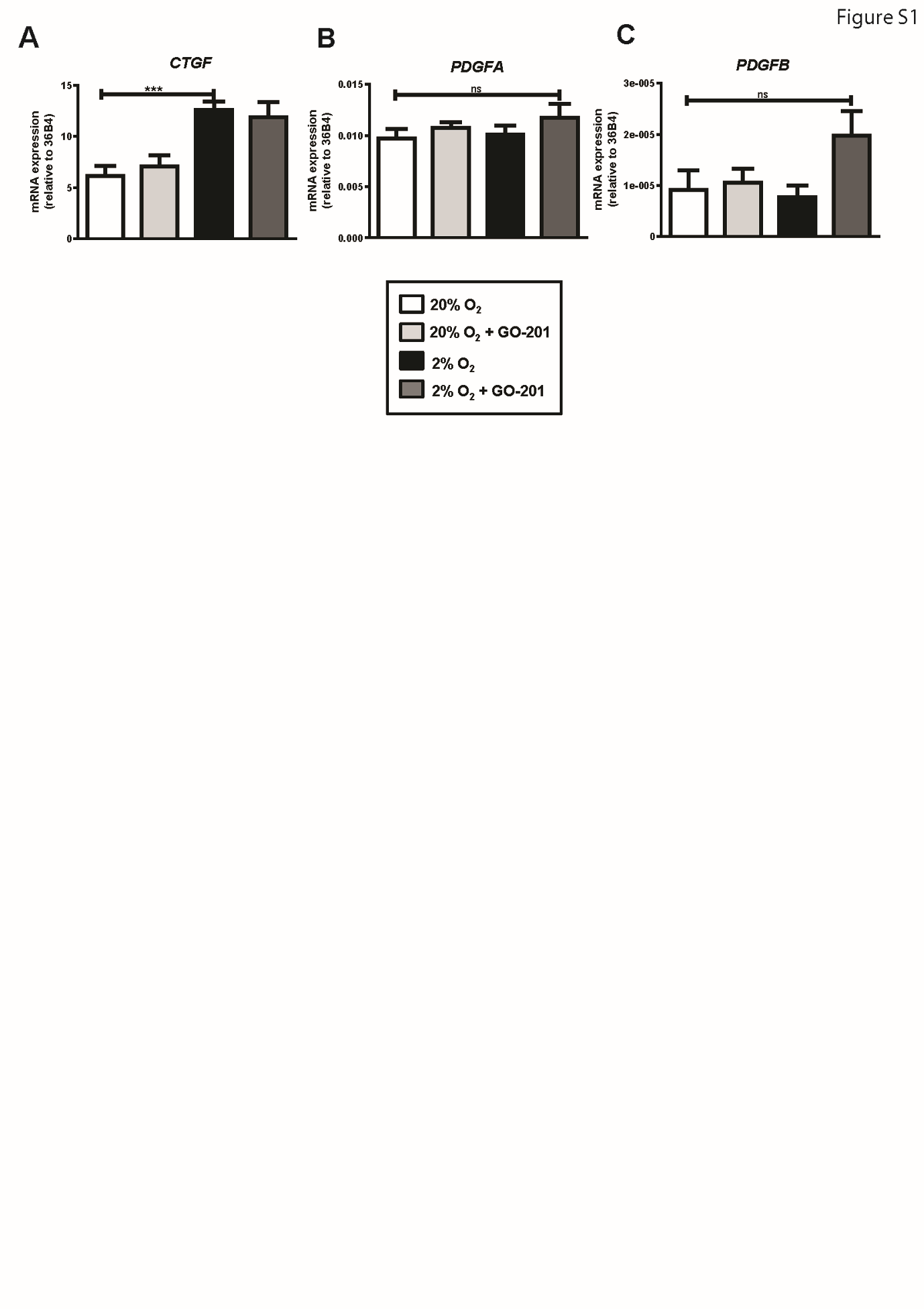
**

**Figure S1.** Effect of hypoxia and/or MUC1-CT inhibition on *CTGF, PDGF-A* and *PDGF-B*.

Gene expression of *CTGF, PDGF-A* and *PDGF-B* on day 11 of osteogenic differentiation of human MSC under 20% or 2% oxygen with or without GO-201. Ribosomal phosphoprotein 36B4 (***p<0.001, ns=not significant). Bars represent averages±SD.

**
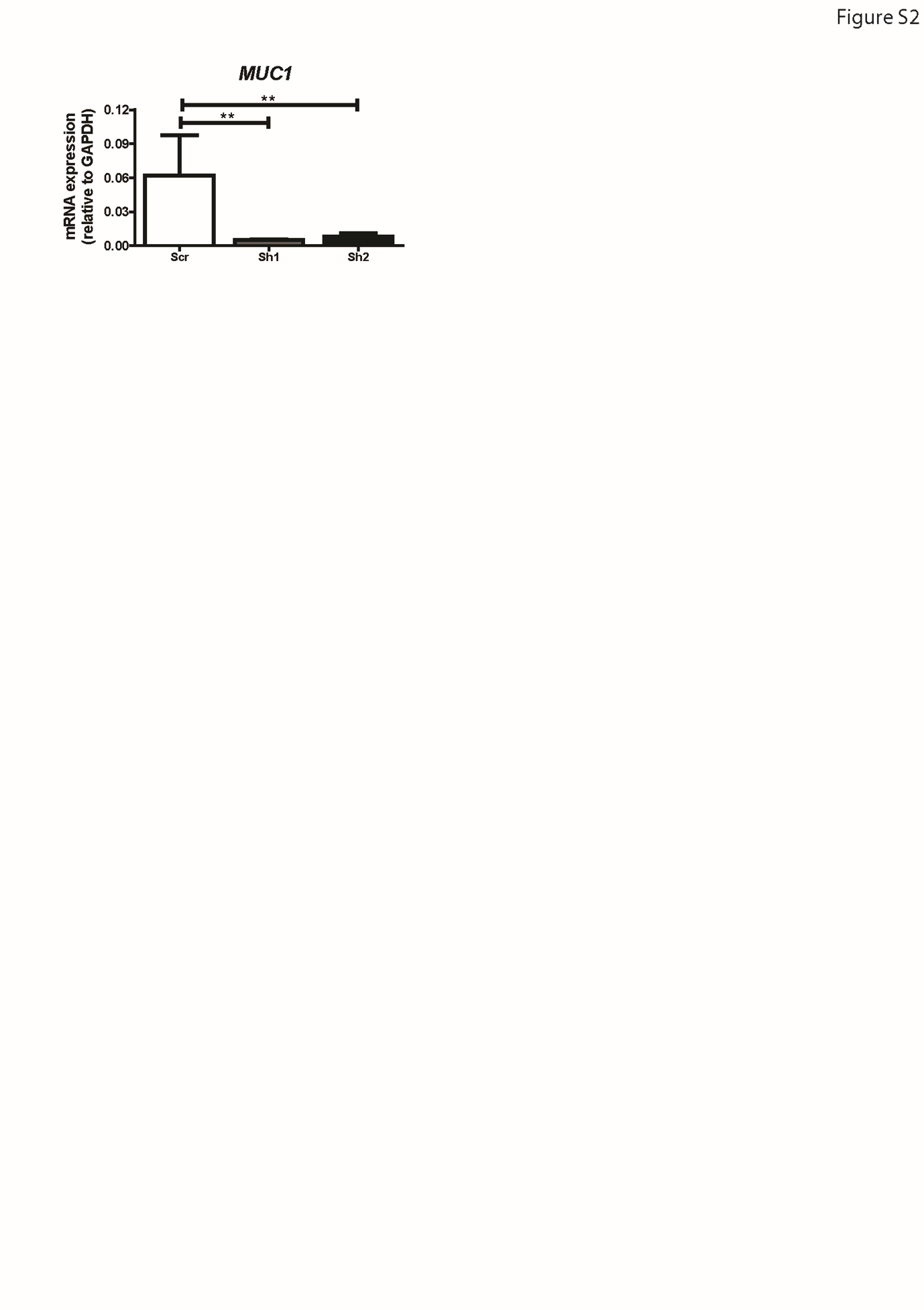
**

**Figure S2.** Assessment of *MUC1* inhibition efficiency of MUC1 sh-RNAs.

Gene expression of *MUC1* in human MSC at day 10. Glyceraldehyde 3-phosphate dehydrogenase (GAPDH) was used as housekeeping gene (**p<0.005). Bars represent averages±SD.

**
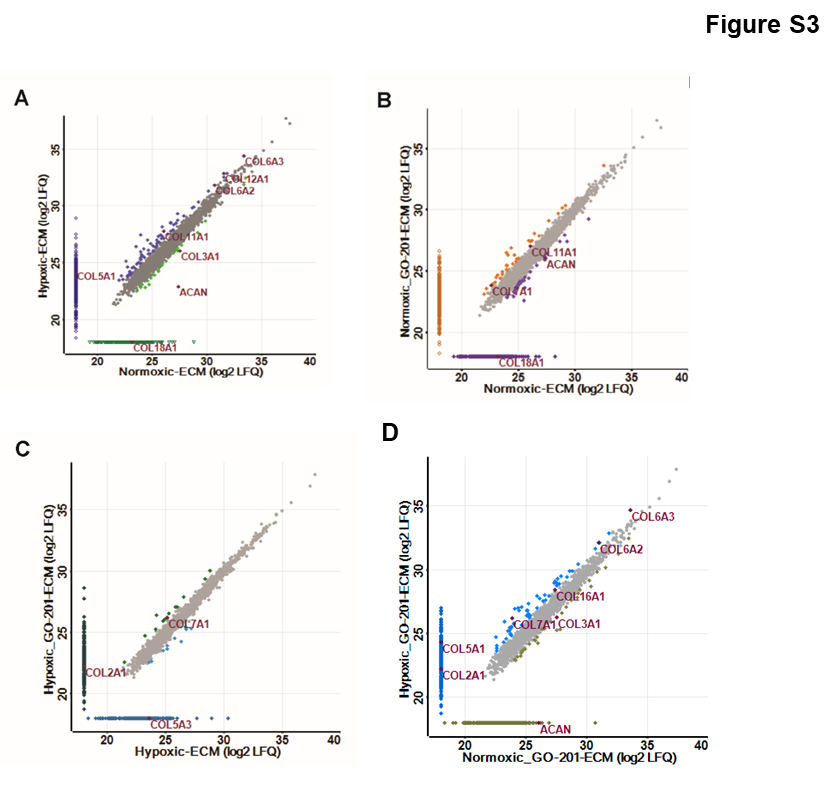
**

**Figure S3.** Comparative effect of normoxia with MUC1-CT inhibition and hypoxia on proteomic landscape of ECM of osteogenic differentiation of MSCs.

**A)** Scatter plots of proteins detected in hypoxic ECM versus normoxic ECM: 390 proteins were only present in hypoxic ECM (dark blue) and 285 proteins were only present in normoxic ECM (dark green); 70 proteins were upregulated (light blue) and 25 proteins were downregulated (light green) due to hypoxia by two or more than two-fold. **B)** Scatter plots of proteins detected in normoxic-GO-201 ECM versus normoxic ECM: 338 proteins were only present in normoxic-GO-201 ECM (dark orange) and 255 proteins were only present in normoxic ECM (dark violet); 31 proteins were upregulated (light orange) and 30 proteins were downregulated (light violet) due to GO-201 by two or more than two-fold. **C)** Scatter plots of proteins detected in hypoxic-GO-201 ECM versus hypoxic ECM: 298 proteins were only present in hypoxic-GO-201 ECM (dark green) and 268 proteins were only present in hypoxic ECM (dark cyan); 12 proteins were upregulated (light green) and 11 proteins were downregulated (light cyan) due to hypoxic-GO-201 treatment by two or more than two-fold. **D)** Scatter plots of proteins detected in normoxic-GO-201 ECM versus hypoxic ECM: 317 proteins were only present in normoxic-GO-201 ECM (dark cyan) and 338 proteins were only present in hypoxic ECM (dark green); 24 proteins were upregulated (light cyan) and 59 proteins were downregulated (light green) due to normoxic-GO-201 treatment compared to hypoxia by two or more than two-fold.

**Supplementary tables:**

**Table S1.** pLKO.1 plasmid-based shRNA sequences against *MUC1*

**shRNA Target Sequence in *MUC1* gene**

Sh-1 CCAGTTTAATTCCTCTCTGGA

Sh-2 GACACAGTTCAATCAGTATAA

**scrambled Oligo sequence**

Scr CCGGCAACAAGATGAAGAGCACCAACTC-

GAGTTGGTGCTCTTCATCTTGTTGTTTTT

**Table S2.** Details of antibodies used for immunoblotting

| **Table S1** | | | | |
| --- | --- | --- | --- | --- |
| **S.No** | **Reagent** | **Dilution** | **Source** | **Identifier** |
| 1 | Rabbit polyclonal anti-Hif1a | 1:1,000 | Cell Signaling Technology | Cat #3716 |
| 2 | Mouse monoclonal anti-vinculin | 1:1,000 | Merck Milipore | Cat # CP74 |
| 3 | Rabbit polyclonal anti-MUC1 | 1:1,000 | Sigma Aldrich | Cat #SAB2108549 |
| 4 | Rabbit monoclonal anti-alpha tubulin | 1:1,000 | Cell Signaling Technology | Cat #2125 |
| 5 | Rabbit monoclonal anti-beta tubulin | 1:1,000 | Cell Signaling Technology | Cat #2146 |
| 6 | Rabbit polyclonal anti-phospho AMPK | 1:1,000 | Cell Signaling Technology | Cat #2531 |
| 7 | Rabbit polyclonal anti-AMPK | 1:1,000 | Cell Signaling Technology | Cat #2532 |
| 8 | Rabbit polyclonal anti-phospho raptor | 1:1,000 | Cell Signaling Technology | Cat #2083 |
| 9 | Rabbit monoclonal anti-raptor | 1:1,000 | Cell Signaling Technology | Cat #2280 |
| 10 | Rabbit polyclonal anti-phospho mTOR | 1:1,000 | Cell Signaling Technology | Cat #2971 |
| 11 | Rabbit monoclonal anti-mTOR | 1:1,000 | Cell Signaling Technology | Cat #2983 |
| 12 | Rabbit polyclonal anti-phospho S6 | 1:1,000 | Cell Signaling Technology | Cat #2215 |
| 13 | Rabbit monoclonal anti-S6 | 1:1,000 | Cell Signaling Technology | Cat #2217 |
| 14 | Mouse monoclonal anti-beta actin | 1:10,000 | Proteintech | Cat #60008-1-Ig |
| 15 | Rabbit monoclonal anti-Phospho-NF-κB p65 | 1:1,000 | Cell Signaling Technology | Cat #3033 |
| 16 | Rabbit polyclonal anti-NF-κB p65 | 1:1,000 | Cell Signaling Technology | Cat #3034 |
| 17 | Mouse monoclonal anti-IkBa | 1:1,000 | Cell Signaling Technology | Cat #4814 |

**Table S3.** Real Time PCR (Q-PCR) primers for SYBR green PCR

| Gene of Interest | Primer Sequence |
| --- | --- |
| 36B4 | FOR: 5’-CGACCTGGAAGTCCAACTAC-3’ |
|  | REV: 5’-ATCTGCTGCATCTGCTTG-3’ |
| MUC1 | FOR: 5’-CCAGCACCGACTACTACCAAGAG-3’ |
|  | REV: 5’-CGTCGTGGACATTGATGGT-3’ |
| VEGFA | FOR: 5’-ACAACAAATGTGAATGCAGACCA-3’ |
|  | REV: 5’-TACCGGGATTTCTTGCGCTT-3’ |
| COL1A1 | FOR: 5’-GACATGTTCAGCTTTGTGGACC-3’ |
|  | REV: 5’-TGATTGGTGGGATGTCTTCGT-3’ |
| COL5A1 | FOR: 5’-CCCAGATGGTGAATACTGGGTC-3’ |
|  | REV: 5’-CTTCGGACTTCTTGTCAGGGA-3’ |
| COL5A3 | FOR: 5’-CCTGCCTCTATCCCGACAAG-3’ |
|  | REV: 5’-GCGTCCACGTAGGAGAACTTCT-3’ |
| TGFβ1 | FOR: 5’-CTACATTTGGAGCCTGGACA-3’ |
|  | REV: 5’-CCGGGTTATGCTGGTTGTA-3’ |
| TGFβ2 | FOR: 5’-GCTTCACCATAAAGACAGGAACCT-3’ |
|  | REV: 5’-TTCTTCACTTTTATTTGGGATGATGT-3’ |
| SMAD2 | FOR: 5’-CCCTCACTCACTGTAGATGGCTTTA-3’ |
|  | REV: 5’-ATAAGCGCACTCCTCTTCCTATATG-3’ |
| SMAD3 | FOR: 5’-ACGCAGGTTCTCCAAACCTATC-3’ |
|  | REV: 5’-CCGGCTCGCAGTAGGTAACT-3’ |
| SMAD4 | FOR: 5’-GGACATTACTGGCCTGTTCACA-3’ |
|  | REV: 5’-ACCAATACTCAGGAGCAGGATGA-3’ |
| SMAD7 | FOR: 5’-CTTAGCCGACTCTGCGAACT-3’ |
|  | REV: 5’-AAATCCATCGGGTATCTGGA-3’ |
| Ki-67 | FOR: 5’-ACGTGAACAGGAGCCAGCACG-3’ |
|  | REV: 5’-AGGCCTTGGAATCTTGAGCTTTCTC-3’ |
| TIE-1 | FOR: 5’-TTCTCATGTGGGCGCGG-3’ |
|  | REV: 5’-CCAGACACGCAAGTCAGGAA-3’ |
| TIE-2 | FOR: 5’-GGAGCGAAAGACCTACGTGAA-3’ |
|  | REV: 5’-GTTCTGTCCTAGGCCGCTTC-3’ |
| ANGPT1 | FOR: 5’-GCCTGATCTTACACGGTGCT-3’ |
|  | REV: 5’-GCATCAAACCACCATCCTCC--3’ |
| ANGPT2 | FOR: 5’-ATGTCCACATCAAACTCAGCTA-3’ |
|  | REV: 5’-GATGCCATTCGTGGTGTGTC-3’ |
| BAX | FOR: 5’-CTGAGCAGATCATGAAGACAGG-3’ |
|  | REV: 5’-CTGCTCGATCCTGGATGAAA-3’ |
| BCL2 | FOR: 5’-AGTACCTGAACCGGCACCT-3’ |
|  | REV: 5’-ACAGTTCCACAAAGGCATCC-3’ |
| CTGF | FOR: 5’-ACGGCGAGGTCATGAAGAAGAACA-3’ |
|  | REV: 5’-TGGGGCTACAGGCAGGTCAGTG-3’ |
| PDGF-A | FOR: 5’-CCGTAGGGAGTGAGGATTCTT-3’ |
|  | REV: 5’-CAGCTTCCTCGATGCTTCTCT-3’ |
| PDGF-B | FOR: 5’-CTGGTCAGCGCCGAGG-3’ |
|  | REV: 5’-ATCTTCCTCTCCGGGGTCTC-3’ |
| APDH | FOR: 5’-CCGCATCTTCTTTTGCGTCG-3’ |
|  | REV: 5’-CCCAATACGACCAAATCCGTTG-3’ |
